# RE1 silencing transcription factor (REST) negatively regulates ALL1-fused from chromosome 1q (*AF1q*) gene transcription

**DOI:** 10.1186/s12867-015-0043-7

**Published:** 2015-09-05

**Authors:** Yuanyuan Hu, Qianwen Sun, Chen Zhang, Qingquan Sha, Xiulian Sun

**Affiliations:** Department of Neurology, Qilu Hospital Shandong University, 107 Wenhuaxi Rd., Jinan, 250012 Shandong Province China; Brain Research Institute, Qilu Hospital of Shandong University, 107 Wenhuaxi Rd., Jinan, 250012 Shandong Province China

**Keywords:** AF1q, REST, Promoter, Gene transcription, Neurodevelopment

## Abstract

**Background:**

ALL1-fused from chromosome 1q (AF1q), originally considered as an oncogenic factor, has been implicated in neuronal development; however, its upstream regulatory mechanisms in neural system remained elusive.

**Results:**

Our study showed that REST (RE1 silencing transcription factor), a key transcription factor in neurodevelopment, could down-regulate the gene expression of *AF1q*. The promoter assay identified a neuron-restrictive silencer element at −383 to −363 bp of human *AF1q* promoter. Electrophoretic mobility shift assay (EMSA) and chromatin immunoprecipitation (CHIP) confirmed the binding of REST to the NRSE in *AF1q* gene promoter. Additionally, the negative correlation between the expression levels of *Af1q* and *Rest* in mice neurodevelopment supported the negative regulation of *AF1q* by REST and the potential functions of AF1q in neurodevelopment.

**Conclusion:**

These results demonstrate that REST regulates *AF1q* gene transcription through directly binding to a NRSE at −383 to −363 bp of *AF1q* promoter.

## Background

The *AF1q* gene was initially identified as a mixed lineage leukaemia (MLL) fusion partner from an infant acute myelomonocytic leukemia patient carrying the t(1;11)(q21;q23) translocation [1], which encoded a highly conserved 90 amino acid residues protein containing a MLLT11 motif and a nuclear export signal [2] with no similarity to other known proteins [1]. An increase in *AF1q* mRNA levels had been shown in leukemic and immature hematopoietic cells [1]. MiR-29b can regulate AF1Q gene expression and lower expression of miR-29b was associated with poor overall survival of acute myeloid leukemia [3]. AF1q had been regarded as an oncogenic factor involved in thyroid oncocytic tumors, breast cancer and testicular germ cell tumors [4–8], though its function had not been well characterized. A recent study showed that AF1Q interacted with T cell factor 7 of Wnt signaling pathway to regulate CD44 and promoted breast cancer metastasis [9]. In addition to the proposed oncogenic role, AF1q had been reported to play an important role in the development of neurons in the peripheral and central nervous systems [10]. The mouse *Af1q*, homologue of human *AF1q*, was found to be significantly up-regulated during the neuronal production from neural stem/progenitor cells [11]. AF1q was differentially expressed during neuronal differentiation [10], but the underlying regulatory mechanisms in neurodevelopment were unknown.

REST (RE1 silencing transcription factor) regulates embryonic and neural stem cells by affecting their derivatives [12–14] and participating in the self-renewal of neural stem cells [13] via regulating the transcription of target genes by binding to a 21-bp DNA element, which is called RE1-binding site/neuron-restrictive silencer element (RE1/NRSE) during neurodevelopment [15]. It was found that the expression of REST was decreased in cultured neurospheres derived from fetal Down syndrome (DS) brain [16] and in the brains of DS mouse models [17]. REST was decreased in Alzheimer’s disease [10, 18]. REST, is expressed throughout early development [19] and acts as transcriptional silencer or activator, which is essential for the regulation of target genes during neuronal development [20, 21]. Downregulation of REST during neurogenesis is necessary for proper neuronal differentiation, while overexpression of REST in differentiating neurons interferes in neuronal gene expression and causes axon guidance errors [22]. REST modulated the expression of genes that were critical in normal neuronal functions including neurotransmitter receptors, synaptic proteins, and ion channels proteins [15, 16]. Recent studies have demonstrated that in contrast to the role of REST in the repression of Rest target genes in in vitro cultured neuronal cells, as well as in non-neuronal cells outside of the brain, the CoRest binding site of Rest is dispensable for embryonic neurogenesis in vivo [12].

In the present study, we demonstrated that *AF1q* transcription could be down-regulated by REST. We also identified *AF1q* promoter sequence and demonstrated that REST was a key transcriptional factor participating in AF1q down-regulation via a NRSE site at −383 to −363 bp of human *AF1q* promoter. Furthermore, our study suggested that the expression of REST and AF1q were negatively correlated during neurodevelopment, implying that AF1Q may be involved in neurodevelopment.

## Results

### REST inhibited human AF1q gene transcription

**Fig. 1:**
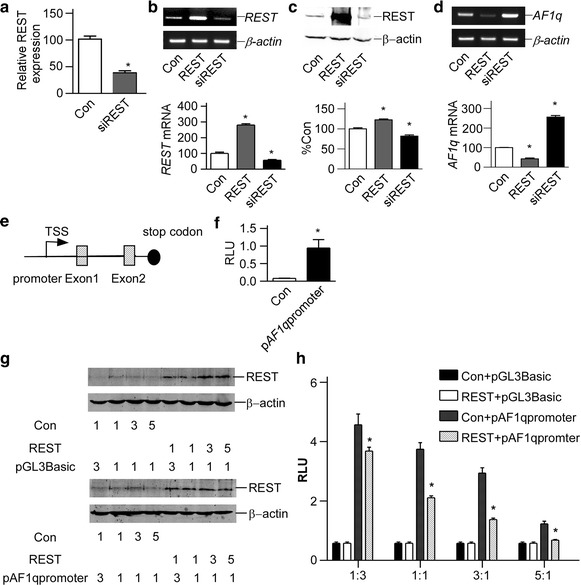
REST inhibited human *AF1q* gene transcription. **a** Real time PCR showed that *REST* expression was decreased by psiREST. SYBR Green gene expression assay of *REST* was performed. 18S rRNA was used as the internal control. The values represent the mean ± SE (*n* = 3). *P < 0.05 by Student’s *t* test. *Con* control. **b** RT-PCR showed that REST expression construct pREST increased *REST* mRNA expression, and REST knock down construct psiREST decreased *REST* mRNA expression. *β-actin* was used as the internal control. The values represented the mean ± SE (n = 3). *P < 0.01 by Student’s *t* test. **c** The knockdown effect of psiREST was verified in protein level by Western blot. REST was detected with anti-REST antibody. β-actin was used as the internal control. The values represented the mean ± SE (n = 3). *P < 0.01 by Student’s *t* test. **d** RT-PCR showed that *AF1q* mRNA was repressed by REST overexpression in HEK293 cells. *β-actin* was used as the internal control. The values represented the mean ± SE (n = 3). *P < 0.01 by Student’s *t* test. **e** Genomic organization of *AF1q* gene. *AF1q* gene had two exons and one intron. TSS, transcription start site. **f** p*AF1q*-promoter contains the functional promoter of *AF1q* gene, containing the 1809-bp fragment from the human *AF1q* gene 5′UTR, was transfected into HEK293 cells. +1 is the first base of the first exon. Dual-luciferase activity was measured by a luminometer 24 h after transfection. The values represented the mean ± SE (n = 3). *P < 0.01 by Student’s *t* test. **g** The expression of REST was examined by Western bolt. REST was detected with anti-REST antibody. Different mass ratios were marked under the Western blot figure. β-actin was used as the internal control. **h** *AF1q* gene promoter was inhibited by REST. REST was co-transfected with p*AF1q* promoter into HEK293 cells. Dual-luciferase activity was measured 24 h after transfection by a luminometer. The values represented the mean ± SE (n = 3). *P < 0.01 by Student’s *t* test

To investigate whether *AF1q* gene transcription was regulated by REST, the endogenous *AF1q* mRNA levels were determined by RT-PCR in cells transfected with REST expression plasmid pREST or its pSuper based silencing vector psiREST. The knock-down efficiency of psiREST was detected by real time PCR using SYBR Green gene expression assay. Silencing plasmid pSuper was performed as control. Real time PCR confirmed that REST expression was reduced by psiREST to 37.57 ± 3.81 % of controls (Fig. 1a). *REST* mRNA level could be elevated or reduced by pREST or psiREST to 280.4 ± 8.331 and 56.81 ± 4.813 % (p < 0.05; Fig. 1a). Similar results were obtained in the protein levels (p < 0.05; Fig. 1b). Overexpression of REST in HEK293 cells could significantly reduce *AF1q* mRNA levels to 42.31 ± 4.567 % of controls (p < 0.01; Fig. 1c). Furthermore, knockdown of REST could markedly increase the *AF1q* mRNA levels to 256.1 ± 8.268 % (p < 0.01; Fig. 1c). The data here indicated that REST was a negative regulator of *AF1q* gene expression.

To further clarify the molecular mechanism of *AF1q* gene transcription, we cloned 1811-bp fragment located in the 5′-flanking region of the human *AF1q* gene (Fig. 1d) into promoterless vector pGL3Basic. TSS represents the first base on exon 1 from Ensemble transcript ENST00000368921, which is 2802-bp upstream of translation start codon. Thus, the expression of luciferase activity in cells transfected with this plasmid depended on the insertion and proper orientation of a functional promoter upstream of the firefly luciferase gene. Plasmid pAF1q promoter (−1349 to +462 bp) was transfected into HEK293 cells, and luciferase activity was measured by a luminometer to reflect *AF1q* promoter activity. Plasmid pAF1q promoter transfected cells had significantly higher luciferase activity compared with controls (0.9445 ± 0.2401 compared with 0.08008 ± 0.006239 relative luciferase units, p < 0.05; Fig. 1e, f), indicating that the 1.8-kb fragment contains the functional promoter region of the human *AF1q* gene.

To investigate whether *AF1q* gene transcription could be regulated by REST, we cotransfected HEK293 cells with REST expression plasmid and p*AF1q* promoter, and examined the promoter activity of human *AF1q* gene. A series of different mass ratio of REST and *AF1q* promoter were selected and screened for modulatory effects on dual-luciferase assay. The transfected mass ratio of REST to *AF1q* promoter was 1:3, 1:1, 3:1 and 5:1, respectively. The REST protein expression was determined by Western blotting (Fig. 1g). Dual-luciferase assay showed that REST overexpression decreased AF1q promoter activity and the effects depended on the mass ratio of REST to AF1q promoter. We could see that the AF1Q promoter activity was reduce by 19.34 ± 8.499, 43.59 ± 6.122, 53.24 ± 6.251, and 44.58 ± 7.531 % with increased expression of REST (p < 0.05; Fig. 1h). These results indicated that the transcription of human AF1q gene could be down regulated by REST.

### Identification of the functional NRSE site in AF1q gene promoter

**Fig. 2:**
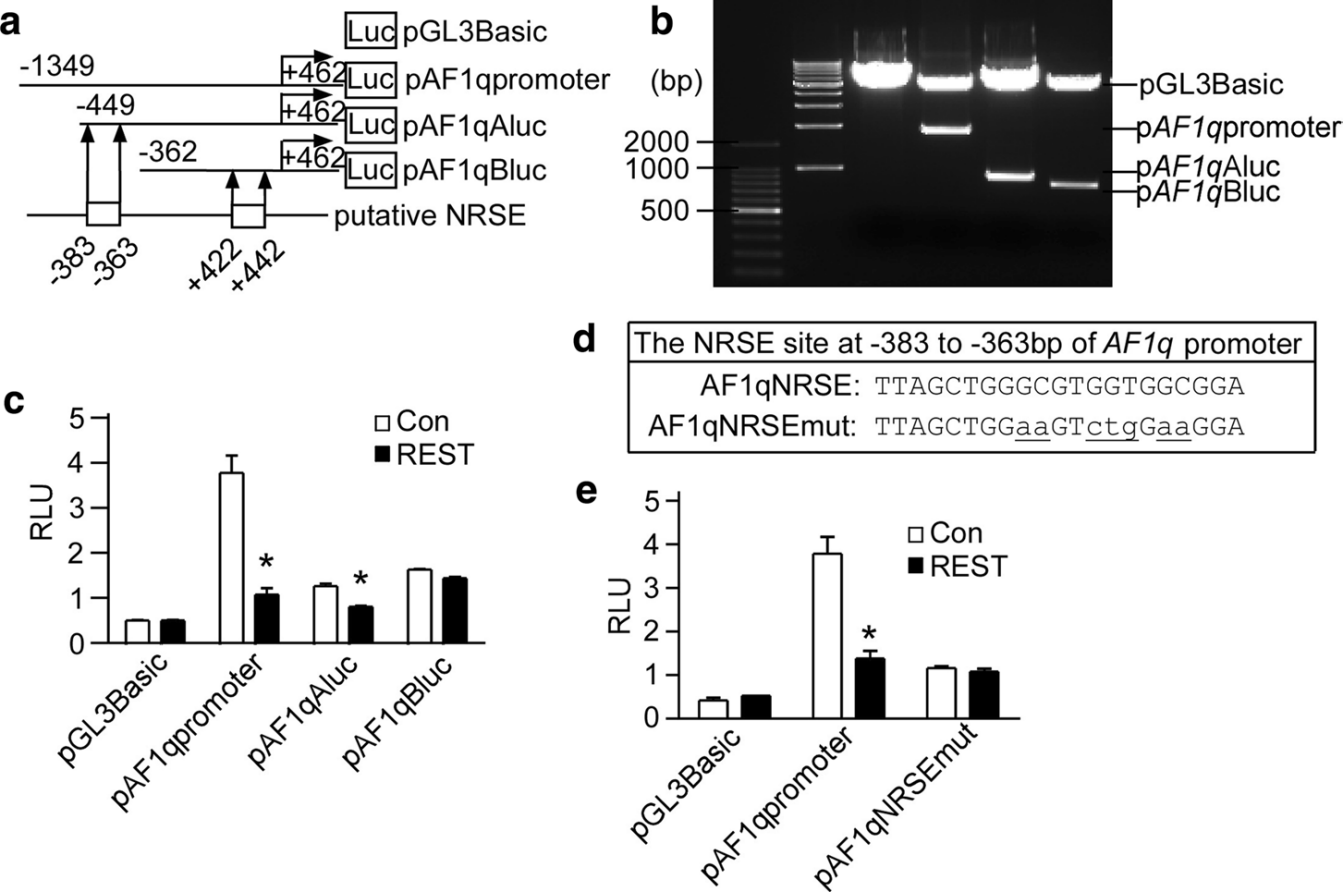
Identification of the functional NRSE site in *AF1q* gene promoter. **a** Schematic diagrams of the *AF1q* promoter deletion constructs consisting of a 5′-flanking region with serial deletions cloned into the promoter-less vector plasmid pGL3-Basic in front of the luciferase reporter gene.* Arrow* indicated the direction of transcription. The numbers represented the end points of each construct. The positions of two putative NRSE sites were shown at the bottom. **b** The deletion plasmids were confirmed by sequencing and restriction enzyme digestion on a 1.5 % agarose gel. Vector size is 4.7 kb, and the *AF1q* gene 5′-flanking fragment inserts ranged from 0.6 to 1.8 kb. **c** HEK293 cells were co-transfected with REST expression vector and various *AF1q* promoter deletion constructs. Plasmid pRL-TK was used to normalize the transfection efficiency, and luciferase activity was measured at 24 h by a luminometer. The values represented the mean ± SE (n = 3). *P < 0.01 by Student’s *t* test. **d** p*AF1q*NRSEmut was made to contain the mutant NRSE site at −383 to −363 of p *AF1q*Aluc, where (5′-TTAGCTGGGCGTGGTGGCGGA-3′) was mutated to (5′-TTAGCTGGaaGTctgGaaGGA-3′). **e** HEK293 cells were co-transfected with REST expression vector and *AF1q* promoter or p*AF1q*NRSEmut. Plasmid pRL-TK was used to normalize the transfection efficiency, and luciferase activity was measured at 24 h by a luminometer. The values represented the mean ± SE (n = 3). *P < 0.01 by Student’s *t* test

Above data showed that *AF1q* gene promoter could be regulated by REST. Previous studied had shown that REST regulated the transcription of neural genes via binding to a NRSE site, a 21-bp consensus DNA sequence, which was present in the regulatory regions of neural genes. In this study, a computer-based transcription factor-binding site search by the JASPAR database revealed that the 1811-bp 5′-flanking region contained two putative NRSE located at −383 to −363 bp and +422 to +442, which were the potential binding sites for REST. To identify the binding site, a series of luciferase reporter gene plasmids controlled by different upstream deletions of human *AF1q* gene promoter region were constructed (Fig. 2a, b). Each deletion construct was transfected into HEK293 cells with REST expression plasmid, respectively. Dual-luciferase assay showed that REST significantly decreased *AF1q* promoter activity of pAF1qAluc, but had no significant effect on pAF1qBluc (Fig. 2c), indicating that the region of −383 to −363 bp of *AF1q* promoter might contain a NRSE. Moreover, the mutation of the NRSE site at −383 to −363 bp of pAF1qAluc abolished the effect of REST (Fig. 2e). These data indicated that the NRSE site located from −383 to −363 bp was essential for REST regulation.

**Fig. 3:**
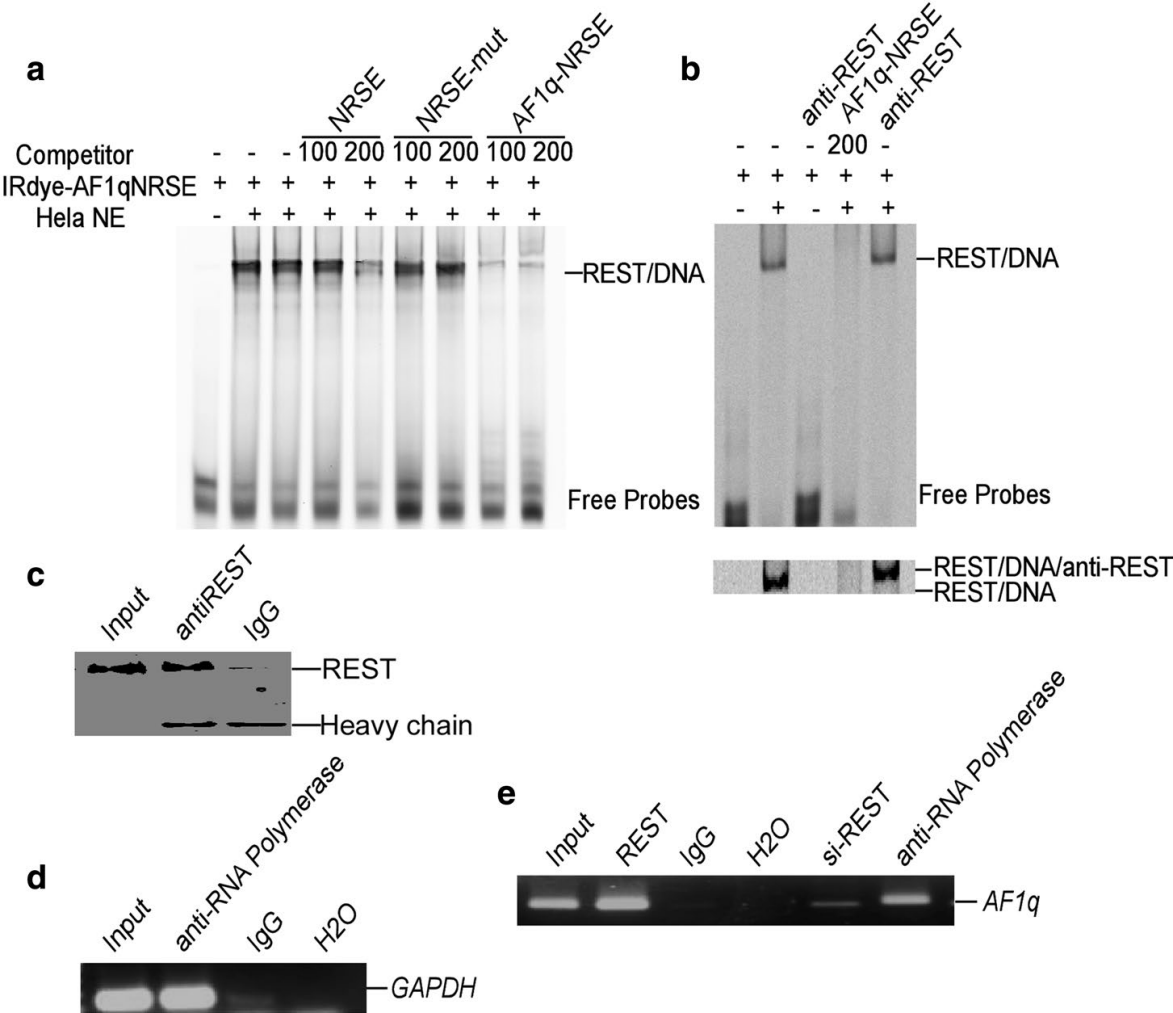
**a** EMSA was performed with IRDye 800-labeled NRSE site of AF1q promoter. Competition assays were performed by further adding different concentration of molar excess of unlabeled competitive oligonucleotides. **b** The addition of anti-REST antibody further shifted DNA–protein complex band.* Lower panel* showed a longer running time. **c** The anti-REST antibody actually immunoprecipitated REST protein.* Lane 1* was input.* Lane 2* was immunoprecipitates by anti-REST antibody.* Lane 3* was immunoprecipitates by IgG antibody. And the lower bands were antibody heavy chain. **d** Anti-RNA polymerase II antibody was used to immunoprecipitate the GAPDH promoter region in CHIP assay in HEK293 cells. A pair of primers targeting GAPDH was used to amplify GAPDH. Signals amplified from input were used as size markers for PCR. IgG and H_2_O were used as negative controls. **e** Anti-REST antibody was used to immunoprecipitate the cross-linked REST-DNA complex in CHIP assay in HEK293 cells. A pair of primers targeting AF1q was used to amplify AF1q-REST. Signals amplified from input were used as size markers for PCR. IgG and H_2_O were used as negative controls. And anti-RNA Polymerase II antibody was used as positive control. Si-REST was from cells transfected with psiREST

To further confirm the NRSE located from −383 to −363 bp in *AF1q* promoter was the binding site for REST, EMSA and ChIP were performed. A 21-bp double-stranded oligonucleotide probe containing the sequence of putative NRSE site in AF1q promoter was synthesized and end-labeled with IRDye 800 infrared dye. Consensus NRSE, mutant consensus NRSE, and the putative NRSE site in *AF1q* promoter were also synthesized as cold probes. A shifted DNA–protein complex band was detected after incubation of the labeled consensus NRSE probe with HeLa cell nuclear extract (lane 2 of Fig. 3a). The binding intensity of the shifted band was significantly reduced by applying 100- or 200-fold molar excess of unlabeled consensus NRSE competitive oligonucleotides (lane 4 and 5 of Fig. 3a). The incubation of 200-fold excessive mutant NRSE oligonucleotides had no competitive effect in the shifted NRSE-REST band (lane 6 and 7 of Fig. 3a). The addition of 100- and 200-fold excess of NRSE-AF1q, corresponding to the NRSE site from −383 to −363 bp of the *AF1q* promoter region, reduced the NRSE-REST shifted band in a dosage-dependent manner (lane 8 and 9 of Fig. 3a). The supershift band with the addition of anti-REST antibody further confirmed the specific binding between REST and AF1Q-NRSE site (Fig. 3b). These results further demonstrated that the NRSE-AF1q site at −383 to −363 in human *AF1q* promoter was the binding site of REST.

To confirm that the NRSE-AF1q site verified in vitro can actually bind to REST in vivo, ChIP was employed to pulldown the REST bound DNA. The anti-REST antibody actually immunoprecipitated REST protein as showed in Fig. 3c. Anti-RNA Polymerase II IP was used as positive control and IgG IP was used as negative control for ChIP. Purified DNA was then analyzed by PCR using Control Primers specific for the GAPDH promoter (Fig. 3d). PCR product was observed in the anti-RNA Polymerase II ChIP (lane 2, Fig. 3d), but not in the IgG ChIP (lane 3, Fig. 3d). ChIP-PCR results showed that REST antibody effectively immunoprecipitated the AF1q-NRSE site (lane 2, Fig. 3e). And the ChIP band was greatly reduced in cells with REST knocked down (lane 5, Fig. 3e). AF1q-NRSE PCR product was also observed in the anti-RNA Polymerase II ChIP (lane 6, Fig. 3e) and not in the IgG ChIP (lane 3, Fig. 3e). AF1q promoter specific DNA was also observed in the Input (lane 1, Fig. 3e). Taken together, these data indicated that the NRSE site, corresponding to *AF1q* promoter −383 to −363 bp, was responsible for the down-regulation of *AF1q* gene by REST.

### *Af1q* expression was negatively correlated with *Rest* in mice neurodevelopment

**Fig. 4:**
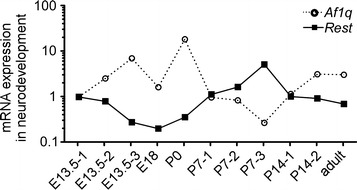
*Af1q* was negatively correlated with *REST* in mice neurodevelopment. **a** Quantitative RT-PCR was performed on cDNA templates prepared from normal mouse brain aging at embryonic days 13.5 (E13.5) and 18.5 (E18.5); at postnatal P1, P7, and P14; and in adult mice. One to three mice were used in each time point as indicated by the numbers after the hyphens. *P* < 0.05 by Spearman correlation test

To investigate whether there are interactions between *Af1q* and *Rest* during neurodevelopment, we detected mRNA levels of *Af1q* and *Rest* in the developing mice brains. RNA was extracted from normal mouse brains aging at embryonic days 13.5 and 18 and postnatal day 1 (P1), P7, P14 and adult. The real-time RT-PCR results showed that mRNA levels of *Af1q* and *Rest* were coordinately expressed with significant negative correlation during neurodevelopment (p = 0.0010, r = −0.8182 by Spearman correlation; Fig. 4a). The plot showed that the E18 measured point was deviated from the statistically supported tendency. These data further demonstrated that *Af1q* gene expression was negatively regulated by Rest in neurodevelopment.

## Discussion

Our study here showed that REST, a key transcription factor in neurodevelopment, can down-regulate the gene expression of *AF1q* through directly binding to a NRSE at −383 to −363 bp of *AF1q* promoter. AF1q, originally considered as an oncogenic factor, is highly expressed in normal hematopoietic tissues, leukemic cell lines and neuronal cells in central nervous system [1, 10, 11, 23]. We can see that the promoter activity in pAF1q promoter is higher than the promoter activity in pAF1qAluc, indicating the region of −1349 to −449 bp containing binding sites for some activators. The decrease of inhibitory effect by REST on pAF1QAluc suggested that the region of −1349 to −449 bp contained binding sites for REST cofactors.

Our study here elucidated the regulatory mechanism of *AF1q* by REST. REST, acting as transcriptional silencer or activator, was essential for the regulation of target genes during neuronal development [20, 21]. REST is required to repress the expression of neuronal genes in undifferentiated neuronal tissue. Expression of REST was highest in embryonic stem cells, but it was decreased while ESCs were differentiated into neuronal stem cells, and it was at low level in mature adult neuronal cells [20]. In addition to participate in neurogenesis, Rest also mediated the interactions between neuron and glia, which was associated with synaptic and neuronal network plasticity and homeostasis in the brain [24, 25]. All these indicated that REST is a key transcription factor in neurogenesis. As a target gene of REST, how AF1q functions in neurogenesis remains elusive. It will be interesting to examine the function of AF1q in neural stem cells and neuronal differentiation. It was reported that REST expression is a protective factor in aging and is decreased in neurodegenerative diseases such as Alzheimer’s disease [18]. It will be interesting to check the expression level of AF1q in some neurodegenerative diseases such as Alzheimer’s disease.

Overexpression of REST has been found in human medulloblastomas, glioblastoma and neuroblastomas [26], in which REST acted as an oncogene to maintain the stem character of neural cells [27]. REST can also act as a tumor suppressor in carcinomas including lung, breast and colon [27]. Though AF1q is regard as an oncogene, the expression level of AF1q is unknown in these cancers. It will be interesting to examine the expression of AF1Q in these cancers associated with REST dysfunction.

## Conclusions

In summary, the current study provides a molecular model for REST in negative regulation of *AF1q* promoter activity and mRNA expression. These results will help to better understand the role of *AF1q* gene in in neural stem cells and neuronal differentiation.

## Methods

### Plasmids construction

The 5′-upstream region of human *AF1q* gene (−1349 to +462 bp, p*AF1q*-promoter) was obtained by PCR of genomic DNA isolated from BAC-human-rp11 using a pair of primers (5′-CGGCTAGCAGGTCTCCACCCTGTCCCTGC-3′ and 5′-CCCTCGAGTTCCCTCCACCCAGCTCTGGTC-3′). The first base of the first exon is referred as bp +1. Then they were cloned into pGL3basic vector (Promega, Madison, WI) containing firefly luciferase reporter gene. Primers used to generate a series of promoter deletion plasmids were as follows: forward primers, 5′-CGGCTAGCGTCAGGAGTTCCAGACCAGC-3′ (p*AF1q*Aluc), 5′-CGGCTAGCTGTAATCCCAGCTACTTGGG-3′ (p*AF1q*Bluc), and reverse primers: 5′-CCCTCGAGCAGAAATGGCCTTGTTCTCT-3′. The p*AF1q*NRSEmut was constructed from pAF1QAluc in which the NRSE site 5′-TTAGCTGGGCGTGGTGGCGGA-3′ was mutated to 5′-TTAGCTGGaaGTctgGaaGGA-3′. All the constructs were verified by sequencing and restriction enzyme digestions. Human REST siRNA was generated using pSuper vector. The target sequence for human REST siRNA is GCTACAATACTAATCGATA. The two strands of RESTsuper sense (5′-gatccccGCTACAATACTAATCGATAttcaagagaTATCGATTAGTATTGTAGCttttta) and RESTsuper antisense (5′-agcttaaaaaGCTACAATACTAATCGATAtctcttgaaTATCGATTAGTATTGTAGCggg) were annealed and inserted into pSuper plasmid to generate the pSuper-REST construct. The vectors pREST were generated as previously described [28].

### Cell culture, transfection and dual-luciferase assay

HEK293 cells were cultured as previously described [29]. All cells were maintained at 37 °C in an incubator containing 5 % CO_2_. All transfections were carried out with lipofectamine™2000 transfection reagent (Invitrogen) according to the manufacturer’s instructions. Luciferase activity was determined as previously described [21].

### RT-PCR

Total RNA was isolated from mouse brains or cells using TRIzol reagent (Invitrogen). The mRNA of *REST* and *AF1q* was quantified by TOYOBO R SYBR Green gene Expression Analysis kit (TOYOBO, Japan). Primers for real-time quantitative and semi-quantitative PCR were as follows: human *AF1q* (270 bp), forward, 5′-CCGCTCGAGGCCACCATGAGGGACCCTGTGAG-3′, and reverse, 5′-GGGGTACCGAGCAAGTCCAGTTCGAAG-3′; human REST (137 bp), forward, 5′-ACTCATCACGGAGAACGCCC-3′, and reverse, 5′-GAGGCCACATAACTGCACTG-3′; mouse *Rest* (244 bp), forward, 5′-CGAGTCTCAGGAAATTGATGA-3′, and reverse, 5′-GCCGTTACCCACTCACTAATAC-3′; human and mouse *β-actin* (141 bp), forward, 5′-GACAGGATGCAGAAGGAGAT-3′, and reverse, 5′-TGATCCACATCTGCTGGAAGGT-3′. All animal protocols were approved by Shandong University Institutional Animal Care and Use Committee and by the Institutional Ethics Committee on Animal Research of Qilu Hospital.

### EMSA and ChIP

The sense sequences of AF1qNRSE, consensus NRSE and mutant AF1qNRSE were 5′-AAAGATTAGCTGGGCGTGGTGGCGGATGCCTGTA, 5′-TTCAGCACCACGGACAGCGCC, 5′-AAAGATTAGCTGGAAGTCTGGAAGGATGCCTGTA. EMSA and ChIP were performed as previously described [28]. Chip-PCR was performed using the DNA reversed from the cross-linked complex with a pair of primers (NRSE-AF1q:5′-GCCTCCGGTTGTACCACT-3′ and 5′-AGCGATTCTCCTGCCTCA-3′). Control primer specific for human GAPDH were 5′-TACTAGCGGTTTTACGGGCG-3′ and 5′-TCGAACAGGAGGAGCAGAGAGCGA-3′.

### Western blot analysis

Western blot was performed as previously described [21]. Anti-REST antibody was from Millipore (#DAM15).

### Data analysis

All experiments were repeated three to five times. In figures one representative picture was shown; quantifications were from three or five independent experiments. The values represent the mean ± SEM. The data were evaluated for statistical significance with Student’s *t* test analysis.
